# Supplementary material for: Multicenter Real-World Data of Subsequent Chemotherapy after Progression to PARP Inhibitors in a Maintenance Relapse Setting
Source: Cancers (Basel). 2022 Sep 11;14(18):4414. doi: 10.3390/cancers14184414 (PMC9497128; doi:10.3390/cancers14184414)
Supplement: Supplementary file 1 [file cancers-14-04414-s001.zip › cancers-1854082-supplementary.pdf]

**Table S1. Baseline characteristics and previous treatments in PS patients Subsequent Platinum-free regimens.**

| | **Total** *N=7* |
|---|---|
| Age at diagnosis (years), Median [IQR] | 66.4 [61.8;67.9] |
| Histology, n (%): HGSOC | 7 (100.0%) |
| BRCA mutational status, n (%): | |
| Positive | 1 (14.3%) |
| WT | 4 (57.1%) |
| Variants of unknown significance (VUS) | 2 (28.6%) |
| Other HRR genes mut, n (%): | |
| Positive | 1 (14.3%) |
| Pt-based chemo immediately previous to PARPi, n (%): | |
| Pt monotherapy | 1 (14.3%) |
| Pt doublet | 6 (85.7%) |
| Months from last cycle of prior Pt to initiation of Pt immediatly pre-PARPi, n (%): | |
| 6-12m | 2 (28.6%) |
| > 12m | 4 (57.1%) |
| Non-available | 1 (14.3%) |
| Number of previous lines to PARPi (including the immediately previous), Median [IQR] | 2.0 [2.0;3.0] |
| Number of previous lines to PARPi (including the immediately previous), n (%): | |
| = 2 | 4 (57.1%) |
| > 2 | 3 (42.9%) |
| Number of cycles of Pt-based chemo immediately previous to PARPi, Median [IQR] | 6.0 [5.0;6.5] |
| Best response to Pt-based chemo immediately previous to PARPi, n (%): | |
| PR | 6 (85.7%) |
| SD | 1 (14.3%) |
| Months with Pt-based CT treatment, Median [IQR] | 4.2 [3.0;5.1] |
| PARPi, n (%): | |
| olaparib | 2 (28.6%) |
| niraparib | 4 (57.1%) |
| rucaparib | 1 (14.3%) |
| Months with PARPi, Median [IQR] | 7.4 [5.6;8.5] |
| Prior PARPi, n (%): | |
| Yes | 1 (14.3%) |
| No | 6 (85.7%) |
| Months from finishing Pt pre-PARPi to Pt pre-PARPi progression, Median [IQR] | 8.4 [7.2;9.3] |

Pt: platinum

**Table S2. Baseline characteristics and previous treatments in PS patients receiving subsequent platinum according to PFI.**

| | **PFI 6-12 months** ***N=33*** | **PFI >= 12 months** ***N=41*** | **p** |
|---|---|---|---|
| Age at diagnosis (years), Median [IQR] | 59.7 [55.9;67.5] | 61.2 [54.0;69.2] | 0.952 |
| Histology, n (%): | | | 1.000 |
| HGSOC | 31 (93.9%) | 39 (95.1%) | |
| HGEOC | 2 (6.1%) | 2 (4.9%) | |
| BRCA mutational status, n (%): | | | 0.024 |
| Positive | 10 (30.3%) | 25 (61.0%) | |
| WT | 21 (63.6%) | 14 (34.1%) | |
| Variants of unknown significance (VUS) | 2 (6.1%) | 2 (4.9%) | |
| Other HRR genes mut, n (%): | | | 0.081 |
| Positive | 1 (3.4%) | 6 (17.1%) | |
| Pt-based chemo immediately previous to PARPi, n (%): | | | 1.000 |
| Pt monotherapy | 3 (9.1%) | 4 (9.8%) | |
| Pt doublet | 30 (90.9%) | 37 (90.2%) | |
| Months from last cycle of prior Pt to initiation of Pt immediatly pre-PARPi, n (%): | | | 0.546 |
| 6-12m | 15 (45.5%) | 16 (39.0%) | |
| > 12m | 17 (51.5%) | 25 (61.0%) | |
| Non-available | 1 (3.0%) | 0 (0.0%) | |
| Number of previous lines to PARPi (including the immediately previous), Median [IQR] | 2.0 [2.0;3.0] | 2.0 [2.0;3.0] | 0.789 |
| Number of previous lines to PARPi (including the immediately previous), n (%): | | | 0.954 |
| = 2 | 19 (57.6%) | 25 (61.0%) | |
| > 2 | 14 (42.4%) | 16 (39.0%) | |
| Number of cycles of Pt-based chemo immediately previous to PARPi, Median [IQR] | 6.0 [5.0;6.0] | 6.0 [5.0;6.0] | 0.783 |
| Best response to Pt-based chemo immediately previous to PARPi, n (%): | | | 0.061 |
| CR | 4 (12.1%) | 14 (34.1%) | |
| PR | 27 (81.8%) | 24 (58.5%) | |
| SD | 2 (6.1%) | 3 (7.3%) | |
| Months with Pt-based CT treatment, Median [IQR] | 4.7 [3.5;5.6] | 4.6 [3.7;5.1] | 0.683 |
| PARPi, n (%): | | | 0.060 |
| olaparib | 14 (42.4%) | 26 (63.4%) | |
| niraparib | 19 (57.6%) | 14 (34.1%) | |
| rucaparib | 0 (0.0%) | 1 (2.4%) | |
| Months with PARPi, Median [IQR] | 6.5 [5.6;8.3] | 14.7 [11.8;22.2] | <0.001 |
| Prior PARPi, n (%): | | | 0.435 |
| No | 29 (96.7%) | 39 (100.0%) | |
| maybe | 1 (3.3%) | 0 (0.0%) | |
| Months from finishing Pt pre-PARPi to Pt pre-PARPi progression, Median [IQR] | 8.8 [6.8;9.8] | 17.1 [13.6;22.6] | <0.001 |
| Months from finishing Pt pre PARPi to CT post PARPi initiation, Median [IQR] | 9.5 [7.4;10.9] | 19.4 [15.2;23.1] | <0.001 |
| Included in the OREO study, n (%): Yes | 0 (.%) | 5 (100.0%) | . |
| Best response to CT administered after PARPi progression, n (%): | | | |
| CR | 6 (8.1%) | 2 (6.1%) | 4 (9.8%) |
| PR | 25 (33.8%) | 9 (27.3%) | 16 (39.0%) |
| SD | 20 (27.0%) | 10 (30.3%) | 10 (24.4%) |
| PROG | 23 (31.1%) | 12 (36.4%) | 11 (26.8%) |
| OS* | 20.6 (13.6-28.9) | 17.2 (13-NA) | 20.6 (13.2-NA) |
| PFS* | 6.6 (6-9.2) | 6.1 (4.9-9.9) | 7.2 (6.5-10.8) |

Pt: platinum; PFI: progression-free interval

**Table S3. ORR, PFS and OS in PR patients.**

| | Total N=30 | ssq Pt N=13 | ssq non-Pt N=17 |
|---|---|---|---|
| Best response to CT administered after PARPi progression, n (%): | | | |
| PR | 14 (46.7%) | 6 (46.2%) | 8 (47.1%) |
| SD | 5 (16.7%) | 1 (7.7%) | 4 (23.5%) |
| PROG | 11 (36.7%) | 6 (46.2%) | 5 (29.4%) |
| OS | 13 (8-15.4) | 10.1 (6.3-NA) | 14.4 (10.7-NA) |
| PFS | 5.1 (4.4-7) | 4.7 (3.4-NA) | 6.8 (5.1-NA) |

Pt: platinum; ssq: subsequent
